# Whole-Genome sequencing and phylogenomic analyses of *Alternaria japonica* and *A*. *telliensis* (Section *Japonicae*)

**DOI:** 10.7150/jgen.142347

**Published:** 2026-10-09

**Authors:** Roumaissa Barkat, Nabahat Bessadat, Justine Colou, Philippe Simoneau

**Affiliations:** 1 University of Angers, Institut Agro Rennes Angers, INRAe, UMR 1345 IRHS, SFR 4207 QUASAV, Beaucouzé Cedex, 49070, France.; 2 Laboratory of Applied Microbiology, University Oran1 Ahmed Ben Bella, BP 1524, El M'Naouer 31000, Oran, Algeria.

**Keywords:** *Alternaria telliensis*, *Alternaria japonica*, nanopore sequencing, genome assembly

## Abstract

Species of *Alternaria* are necrotrophic fungi responsible for leaf spot diseases on a wide range of cultivated and wild plants. Species delimitation within this genus, however, often relies on morphological features and a limited set of molecular markers, which can constrain phylogenetic resolution at finer taxonomic scales. This is the case for *Alternaria telliensis*, a species recently described from Algeria and subsequently reported in Iran, whose relationships within section *Japonicae* require clarification. To provide a higher resolution framework for this species, we performed whole-genome sequencing, assembly, and BUSCO completeness assessment for type strains of *A. telliensis* (CBS 145643) and *A. japonica* (CBS 118390), using Oxford Nanopore Technologies MinION sequencing. The genome assembly of *A. telliensis* spans 34.4 Mbp across 132 contigs, with an N50 of 2.2 Mbp and a GC content of 50.94%. The *A. japonica* assembly comprises 33.5 Mbp in 196 contigs, with an N50 of 1.6 Mbp and a GC content of 51.47%. Phylogenomic analysis places *A. telliensis* alongside *A. japonica* within section *Japonicae*. These genomic data support the classification of *A. telliensis* and contribute to clarifying taxonomic relationships within the genus *Alternaria*.

## Introduction

*Alternaria telliensis* was originally described from *Solanaceae* in northwestern Algeria and subsequently reported as a causal agent of cabbage leaf spot in Iran 1,2. Preliminary characterization of this species relied on morphological analyses combined with multi-locus phylogenies (ITS, GPD, RPB2, TEF1), which placed it within section *Japonicae*, alongside *A. japonica*. However, when mono-locus phylogenies based on ITS or GPD were applied, *A. telliensis* grouped with members of section *Ulocladioides*.

Whole-genome sequencing provides a higher-resolution framework for addressing the taxonomic ambiguity. Phylogenomic analyses based on single-copy orthologous genes can capture broader evolutionary signal than analyses based on a few loci. The genus *Alternaria* was initially divided into 24 sub-generic sections 3 and now comprises 30 sections and 9 monotypic lineages. More than 280 *Alternaria* genome sequences are publicly available in NCBI database. However, 73% are from members of section *Alternaria* and the remaining are divided into ten additional sections and one monotypic lineage. Unfortunately, no genome sequence from species of section *Japonicae* is currently available although species within this section might represent a threat for Brassicaceae crops.

This study aimed to generate whole-genome assemblies of the type strain of *A. telliensis* (CBS 145643) and *A. japonica* (CBS 118390) and reconstruct their phylogenomic relationships with selected *Alternaria* species.

## Materials and Methods

Genomic DNA was extracted from frozen mycelium cultured for 7 days on Potato Dextrose Agar (PDA) medium on a pectocellulosic membrane after bead grinding, following a high-molecular weight fungal DNA extraction protocol for filamentous fungi 4. DNA quality and purity were assessed using a NanoDrop spectrophotometer and DNA concentration was confirmed by fluorometric quantification using a Qubit dsDNA HS Assay Kit (Thermo Fisher Scientific).

Whole-genome sequencing was performed using the Oxford Nanopore Technologies MinION (ONT) platform with the Native Barcoding Kit V14 (SQK-NBD114.24) and the flowcell FLO-MIN114 with R10.4 pores 5. The basecalling was performed on MinKNOW (v25.03.9) with the Dorado software (v7.8.3). Nanopore reads were filtered for quality and length using NanoFilt with a minimum quality threshold of Q >= 10 and a minimum read length of 1,000 bp. Read quality was assessed using NanoStat and NanoPlot from the NanoPack toolkit 6. De novo assembly was performed with Flye (v2.9.6) using raw long reads, an estimated genome size of 31 Mb and three iterations 7. The initial assemblies were improved through three successive polishing cycles with Racon (v1.5.0) 8. For each polishing cycle, reads were aligned to the assembly using minimap2 (v2.24) in Oxford Nanopore mode 9.

Assembly quality before and after polishing was evaluated using QUAST v5.3.0 10. Assembly completeness was estimated using BUSCO (v6.0.0) against the dothideomycetes_odb10 lineage dataset (3,786 BUSCO groups) 11, using miniprot as the gene predictor. The assemblies of *A. telliensis* and *A. japonica* contained 3,766 (99.5%) and 3,769 (99.6%) complete BUSCOs, respectively. Overall assembly statistics were generated using SeqKit 12.

Structural gene prediction was performed using Funannotate v1.8.17. The soft-masked genome assembly was used as input. Protein evidence was generated by aligning a reference protein database containing 560,728 proteins against the genome using DIAMOND and Exonerate. Ab initio gene predictors (Augustus, GlimmerHMM, and SNAP) were trained on BUSCO derived gene models (fungi_odb10 lineage dataset) identified within the assembly. Gene model consensus was generated using EvidenceModeler (EVM), combining predictions from Augustus (weight = 1), high-quality Augustus predictions (weight = 2), GlimmerHMM (weight = 1), and SNAP (weight = 1). Gene models shorter than 50 amino acids, spanning assembly gaps, or corresponding to transposable elements were removed. tRNA genes were predicted using tRNAscan-SE.

A comparative genomic dataset was constructed using selected *Alternaria* genome assemblies retrieved from the NCBI GenBank database. The dataset included representatives of ten *Alternaria* sections (plus one monotypic lineage) and *Stemphylium lycopersici* (GCA 003268315.1) as outgroup. The assemblies used for comparison are listed in Table S1. All comparative analyses were performed on the concatenated alignment of 5,399 single-copy orthologous genes in the comparative dataset. Orthologous gene clustering was performed using OrthoFinder (v2.5.2) 13. Protein sequence similarity searches were conducted with DIAMOND 14, and orthogroup delineation used Markov clustering of protein similarity relationships 15. Multiple sequence alignments were generated for each inferred orthogroup using MAFFT v7 16.

The species phylogeny was reconstructed under the maximum-likelihood framework using IQ-TREE v2.2.2.7 17. The best-fit substitution model was Q.plant+F+I+R8, selected according to the Bayesian Information Criterion in ModelFinder 18. Branch support was assessed using ultrafast bootstrap analysis with 1,000 replicates and the SH-aLRT test with 1,000 replicates 19,20.

## Results and Discussion

Nanopore read distributions were visualized for *Alternaria telliensis* and *A. japonica* using NanoPlot (Figure S1). The Nanopore read dataset for *A. telliensis* contained 382,467 reads and 1,260,768,324 bases, whereas the *A. japonica* dataset contained 570,917 reads and 1,780,512,442 bases. Estimated sequencing coverages were approximately 36.6x for *A. telliensis* and 53.1x for *A. japonica* (Table 1). The two datasets had similar mean read qualities, with Q22.5 for *A. telliensis* and Q22 for *A. japonica.*

The *A. telliensis* genome assembly had a total length of 34,408,234 bp distributed across 132 contigs, with a GC content of 50.94%. Its largest contig was 3,841,795 bp, and its N50 and N90 were 2,186,759 bp and 525,007 bp, respectively. The *A. japonica* assembly had a total length of 33,509,493 bp distributed across 196 contigs, with a GC content of 51.47%. Its largest contig was 5,995,486 bp, and its N50 and N90 were 1,655,298 bp and 351,347 bp, respectively. BUSCO analyses revealed highly complete assemblies, the *A*. *telliensis* genome contained 99.5% complete BUSCOs, whereas the *A*. *japonica* genome contained 99.6% complete BUSCOs. Neither assembly contained ambiguous bases, with N's per 100 kbp equal to zero. These results are consistent with what has been reported for species of the genus *Alternaria* whose genome sizes varied from 31 Mbp (*A. brassicicola*) to 40 Mbp (*A. metachromatica*) with an estimated number of genes varying from 10,000 (*A. dauci*) to 13,000 (*A. linariae*) 21.

The maximum-likelihood phylogenomic tree reconstructed from the concatenated alignment of single-copy orthologous genes across the comparative dataset was consistent with the established sectional classification within the genus *Alternaria*, with all major sections recovered as well as the monotypic lineage *A. brassicae*. *A. telliensis* and *A. japonica* were placed within section *Japonicae* with maximum bootstrap support (100%) (Figure 1). This placement supports the assignment of *A. telliensis* to section *Japonicae*.

This study constitutes the first genome sequence report for two *Alternaria* species of section *Japonicae* that are both considered dangerous for Brassicaceae plants*.* Although* A. japonica* has been reported worldwide, records of *A. telliensis* are recent and, up to now, restricted to Algeria and Iran. Future comparative studies with available genome sequences from other important pathogens of Brassicaceae such as *A. brassicae* and *A. brassicicola* may contribute to identify key determinants of pathogenicity for these *Alternaria* species.

## Supplementary Material

Supplementary figure and table.

## Figures and Tables

**Figure 1:**
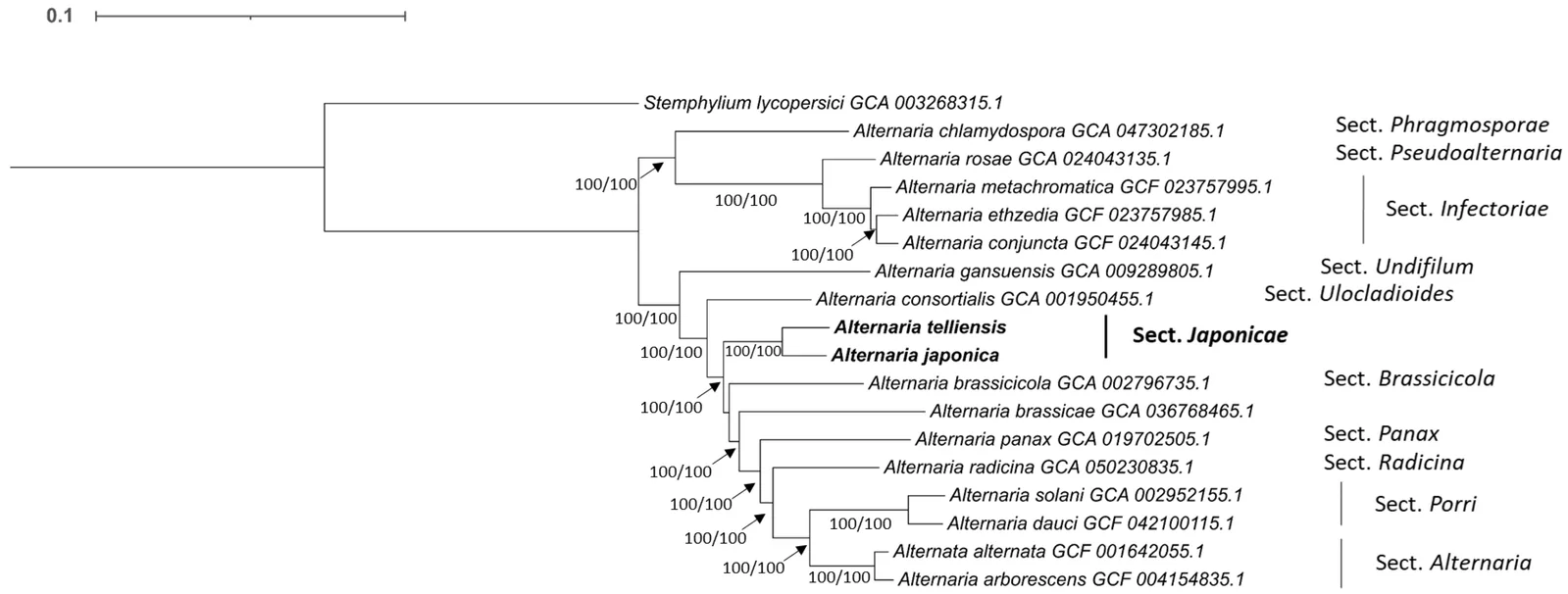
Phylogenomic tree constructed by the maximum likelihood (ML) method from the concatenated alignment of single-copy orthologous genes, illustrating the relationships between the different species of the *Alternaria* sections. *Stemphylium lycopersici* (GCA 003268315.1) was used as outgroup. Numbers at nodes indicate SH-aLRT support (%) / ultrafast bootstrap (UFBoot) support (%), based on 1,000 replicates each.

**Table 1:** Assembly statistics for *Alternaria telliensis* and *A. japonica*.

Strain	Contigs	Largest contig (bp)	Total length (bp)	GC content (%)	N50 (bp)	Coverage(x)	Number of predicted genes
*A. japonica*	196	5,995,486	33,509,493	51.47	1,655,298	53.1	9,522
*A. telliensis*	132	3,841,795	34,408,234	50.94	2,186,759	36.6	9,703

## Data Availability

This Whole Genome Shotgun project has been deposited in DDBJ/ENA/GenBank under the accession no. JCBLRW000000000 and JCBLRX000000000 for *A. japonica* CBS 118390 and *A. telliensis* CBS 145643, respectively. The versions described in this paper are the first versions.
