# Supplementary material for: Whole-Genome sequencing and phylogenomic analyses of Alternaria japonica and A. telliensis (Section Japonicae)
Source: J Genomics. 2026 Oct 9;14:30–3. doi: 10.7150/jgen.142347 (PMC13654648; doi:10.7150/jgen.142347)
Supplement: Supplementary file 1 [file jgenv14p0030s1.pdf]

Table S1 : Genome assemblies used in the comparative phylogenomic analysis.

Species	Sections	Code	N° accession GenBank	Sequencing method	Genome size (Mb)	Contigs	Contig N50	GC%
Alternaria chlamydospora	*Phragmosporae*	SC11	GCA 047302185.1	Oxford Nanopore ; Illumina NovaSeq	36.4	19	3.3 Mb	51.5
Alternaria rosae	*Pseudoalternaria*	BMP 2777	GCA 024043135.1	Illumina HiSeq	32.8	203	794.6 kb	52
Alternaria metachromatica	*Infectoriae*	BMP 0045	GCF 023757995.1	Illumina HiSeq	37.3	12	1 Mb	50
Alternaria ethzedia	*Infectoriae*	BMP 0044	GCF 023757985.1	Illumina HiSeq	36	13	863.8 kb	50.5
Alternaria conjuncta	*Infectoriae*	BMP 0040	GCF 024043145.1	Illumina HiSeq	36.1	179	1.9 Mb	50.5
Alternaria guansuensis	*Undifilum*	LYZ1412	GCA 009289805.1	Illumina	75.1	1,034	163.8 kb	37
Alternaria consortialis	*Ulocladioides*	JCM 1940	GCA 001950455.1	HiSeq 2500	34.2	105	1 Mb	50.5
Alternaria brassicicola	*Brassicicola*	Abra43	GCA 002796735.1	PacBio	31	29	2.1 Mb	51
Alternaria brassicae	/	PNT	GCA 036768465.1	PacBio	33.8	10	3.5 Mb	51
Alternaria panax	*Panax*	BNCC115425	GCA 019702505.1	PacBio ; Illumina HiSeq	34.8	42	2.8 Mb	51
Alternaria radicina	*Radicina*	CBR2	GCA 050230835.1	Illumina NovaSeq ; Oxford Nanopore	34.6	6	6.5 Mb	49.5
Alternaria solani	*Porri*	NL03003	GCA 002952155.1	PacBio	32.8	10	2.9 Mb	51.5
Alternaria dauci	*Porri*	A2016	GCF 042100115.1	PacBio Sequel II ; Illumina NovaSeq	34.9	10	3.4 Mb	50.5
Alternaria alternata	*Alternaria*	SRC1lrK2f	GCF 001642055.1	Illumina	33	91	1.1 Mb	51.5
Alternaria arborescens	*Alternaria*	FERA 675	GCF 004154835.1	Illumina MiSeq	33.9	328	862.6 kb	51
Stemphylium lycopersici	/	CIDEFI 212	GCA 003268315.1	Illumina HiSeq	34.1	633	236.5 kb	51.5

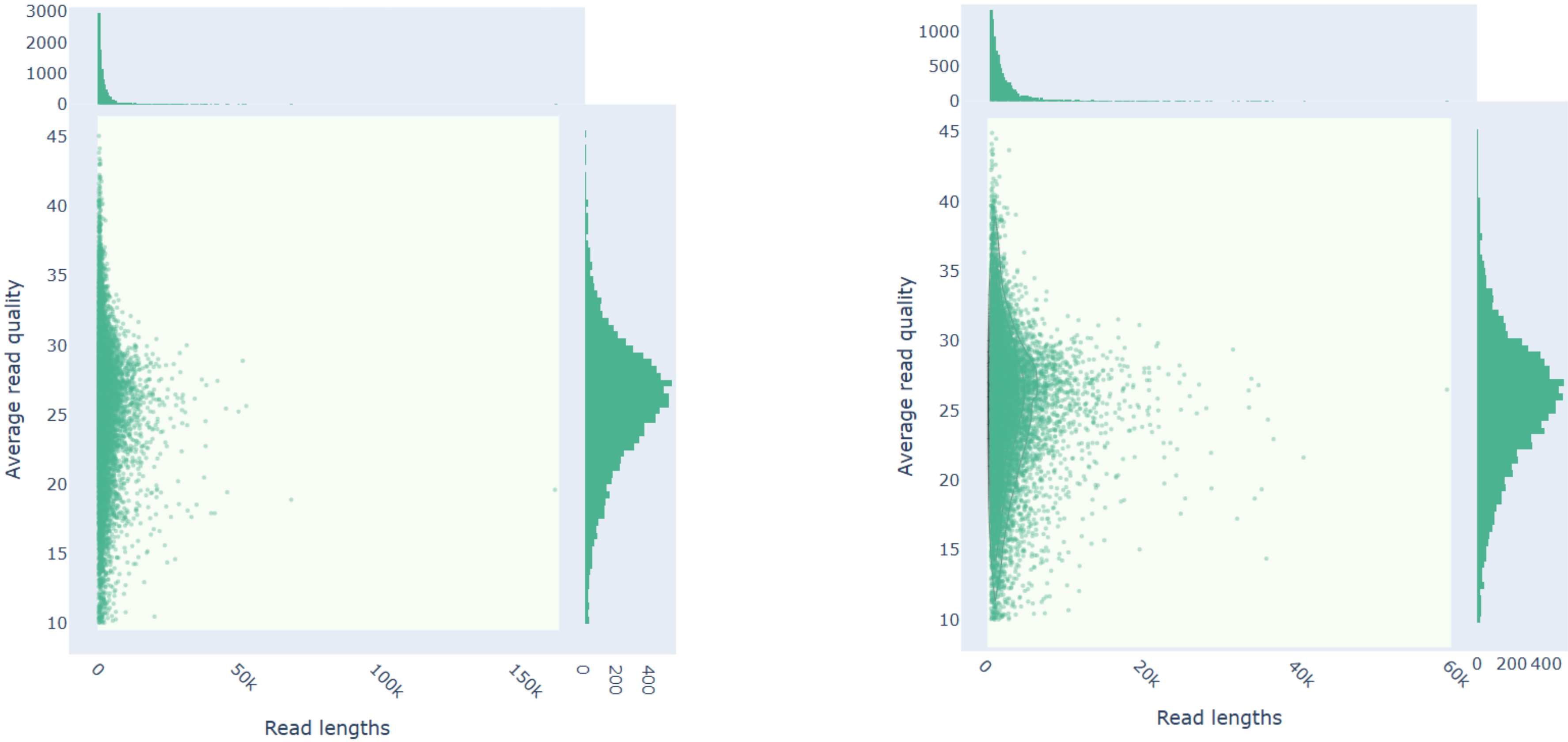

Figure S1 Scatter plot of Nanopore readings for *A. telliensis* strain CBS145643^{T} (left) and *A. japonica* strain CBS118390^{T} (right), showing reading length as a function of quality score, generated with NanoPlot (kde graph).
